# Exploring the Impact of Pickleball for Improving Mood in First-Year University Students—A Pilot Study in Japan

**DOI:** 10.3390/jfmk10030352

**Published:** 2025-09-14

**Authors:** Max Nghiem Lee, Michael Benjamin Fung, Goichi Hagiwara

**Affiliations:** 1 School of Humanities and Sciences, Stanford University, Building 1, Main Quad 450 Jane Stanford Way, Stanford, CA 94305-2070, USA; maxnlee@stanford.edu (M.N.L.); mikefung@stanford.edu (M.B.F.); 2 Department of Sport Science and Health, Faculty of Human Science, Kyushu Sangyo University, 2-3-1 Matsukadai, Higashi-ku, Fukuoka 813-8503, Japan

**Keywords:** Pickleball, mood change, university students, gender differences, physical activity content

## Abstract

**Background**: Pickleball has gained global popularity as a socially engaging and accessible sport, but little is known about its short-term psychological effects on younger populations, particularly university students. This pilot study examines whether participation in a pickleball class during a university course produces immediate mood improvements among Japanese first-year students. **Methods**: A total of 106 sports science students (75 men and 31 women; *M* = 18.44, *SD* = 0.55) participated in a 100 min pickleball tournament. Mood states were measured pre- and post-activity using a Two-Dimensional Mood Scale (TDMS) to assess vitality, stability, pleasure, and arousal. A mixed-design ANOVA (time × gender) was used for analysis. **Results**: A significant main effect of time was found for vitality (*F*(1,103) = 4.97, *p* = 0.028, *η*^2^ = 0.046), indicating improved vitality after participation. Other mood indices showed positive but non-significant trends (pleasure: *p* = 0.127; arousal: *p* = 0.067; stability: *p* = 0.812). No significant main effects of gender or time × gender interactions were observed. Qualitative responses supported these findings, with 64% of participants describing the activity as “fun” or “good” and 24% referencing social themes such as “cooperation” and “exchange”. **Conclusions**: Short-term participation in pickleball during a university class improved vitality and fostered social enjoyment among first-year students, with broadly similar benefits for men and women. As a pilot study, the findings highlight pickleball’s potential as a low-barrier, socially interactive activity to support students’ mental health in educational settings, although future studies with diverse samples and controlled designs are needed.

## 1. Introduction

In recent years, the increasing global popularity of pickleball has attracted the attention of researchers, not only as a recreational activity but also as a potential practical option within the broader context of leisure sports. Because pickleball offers easy accessibility, a social environment, and low-impact physical demands, it can be played by individuals across a wide range of backgrounds and ages. For these reasons, pickleball has become a popular sport enjoyed by all generations worldwide. Although the absolute number of pickleball participants in Japan (approximately 30,000 as of 2025) [1] is small relative to the national population, participation has increased approximately five-fold within a single year, suggesting rapid growth and emerging social relevance in university and community settings [2].

Physical activity is a fundamental component of overall health and well-being, with extensive research demonstrating its importance in both physical and mental health outcomes. Current literature consistently shows that regular physical activity benefits human health in several ways, such as improving cardiovascular function, reducing depressive symptoms, and enhancing mood. Other studies about exercise have also associated physical activity with immediate mood enhancement and emotional regulation from pre- to post-activity, making physical activity a valid tool for mental wellness in daily life [3]. However, research on the acute mood effects of individual exercise remains limited because most existing literature focuses on long-term health benefits. Therefore, there is insufficient evidence regarding the short-term benefits of physical activity, particularly among university students in Japan.

Recent systematic reviews further highlight that incorporating physical activity into university programs can improve mental health, retention, and academic outcomes among higher education students [4,5,6]. This suggests that examining the psychological effects of pickleball in first-year students has both theoretical and practical significance.

Pickleball has gained recognition as a socially engaging and accessible sport. Physical therapist and researcher Sandra Webber categorized pickleball as a moderate-to-vigorous-intensity activity. A typical pickleball session lasting 60–90 min allows players to burn more than 500 calories, whether playing singles or doubles [7]. According to Webber, participating in two to three sessions per week is sufficient for individuals to achieve the World Health Organization’s recommendation of 150 min of moderate activity per week [8]. Building on these findings, Denning et al. conducted a comparative study that showed that playing pickleball doubles provides greater cardiovascular benefits than leisurely walking. This study found an increase in heart rate (14%), caloric expenditure (36%), rate of perceived exertion (44%), and levels of enjoyment (150%) [9]. Extending these findings, Smith et al. [10] found that middle-aged and older adults who played pickleball for one hour, three days per week for six weeks, experienced significant improvements in blood pressure, cholesterol levels, and cardiorespiratory fitness.

Physical activity improves cardiovascular health and contributes significantly to mental health. Studies conducted across diverse populations have linked regular physical activity to reduced depression and improved mood, particularly in older adults. While these effects are generally well-established for physical activity, pickleball may offer unique advantages because it combines accessibility, moderate intensity, and social interaction, making it a particularly practical option in university settings. Pickleball offers both physical exercise and social interaction. Unlike traditional sports, pickleball can be learned quickly even by beginners, allows mixed-gender participation, and has the potential to enhance a sense of accomplishment and social connections. In a study by Heo et al. [11], 153 older adult pickleball players who played regularly showed a significant negative correlation between pickleball participation and depression levels, suggesting that frequent engagement in pickleball may help reduce depressive symptoms. A meta-analysis by Netz et al. [3] also supports this connection, highlighting studies that examined the effects of physical activity programs on the psychological well-being of older adults.

Previous studies have largely focused on older adults and long-term programs. Whether such psychological benefits extend to younger populations and can be observed after a single, short-term session remains unclear. University students face unique developmental and academic stressors that make them an important group for investigation. Therefore, this study aims to examine immediate mood changes among first-year Japanese university students following their participation in a pickleball class conducted as part of a regular physical education course. As a pilot study, its purpose is to evaluate the feasibility and short-term psychological effects rather than to provide generalized causal claims.

This study is unique not only in focusing on pickleball but also in being conducted within the context of an actual university physical education class. Rather than a strictly controlled laboratory experiment, it aims to evaluate the feasibility and psychological effects of pickleball in a real educational environment, thereby providing practical insights into how the sport can be integrated into student wellness programs. Pickleball was chosen for this context because it satisfies the requirements for moderate-to-vigorous physical exercise while also incorporating a social interaction component, making it particularly suitable for university students.

## 2. Materials and Methods

In line with the aim of this pilot study, which is to evaluate short-term psychological effects of pickleball in an actual educational setting, an appropriate assessment tool was required to capture immediate changes in mood states. Using pickleball to examine mood states requires a sufficiently sensitive measure to detect rapid psychological fluctuations. Traditional mood measures often focus on stable and long-term affective states, but they lack sensitivity in detecting rapid mood fluctuations during participation in physical sports [4]. The Two-Dimensional Mood Scale (TDMS), developed by Sakairi and Nakatsuka, offers a reliable solution to this research challenge. Their model was designed to assess and regulate individuals’ momentary mood states, making it an ideal foundation for our investigation into pickleball’s mood effects [12].

In addition to using this scale as a measurement tool, our study also compared its findings to those reported in Sakairi and Nakatsuka’s original research in order to identify specific effects on the shared mood metrics and situate the present pilot study within the established literature. TDMS represents a significant advancement in momentary mood assessment, allowing individuals to monitor and modulate their psychological states in real time. Sakairi et al. [12] summarized that this approach is based on the dimensional theories of affect, which aim to differentiate mood from emotion and emphasize two fundamental axes: pleasure and arousal. Represented within a two-dimensional mapping system, the model enables researchers to represent any mood state at a distinct point in the mapping space. Therefore, individuals can plot their goals and measure the distances from them over time. This framework provides diagnostic clarity and clear directional guidance for understanding and supporting mood regulation. While the original framework can be used to assess both long- and short-term changes in mental states, our study focuses specifically on acute momentary states measured before and after the activity.

The scale operationalizes this theoretical model through four distinct affective quadrants. The reliability and variability of this dimensional approach have been confirmed in previous studies by Sakairi and Nakatsuka, establishing its psychometric validity for momentary mood assessments. High arousal-pleasure (vitality): characterized by energetic, lively states. Low arousal-pleasure (stability): encompasses relaxed, calm states. High arousal–displeasure: including nervous, irritated states. Low-arousal displeasure: covering lethargic, mentally sluggish states [12].

To examine the psychological effects of pickleball in practice, a doubles pickleball tournament was conducted during a 100 min university class session that began at 11:00 a.m. The tournament included 12 teams with 106 first-year college students, with approximately nine students per team. All participants were first-year students enrolled at a Japanese university. All teams were formed based on small groups that had been randomly formed in advance during the first-year compulsory seminar at the beginning of the semester. Participants ranged in age from 18 to 20 years and included 75 males and 31 females (age *M* = 18.44, *SD* = 0.55). Most of the students had experience in competitive sports but little or no experience with pickleball.

The tournament used a single-elimination format, including the first round, quarterfinals, semifinals, and finals, and rankings were determined up to second place, with no third-place playoffs. Each match followed a best-of-three-sets format, with each set lasting five minutes. Player substitutions were allowed between sets. Up to eight courts were used to allow multiple matches to occur simultaneously. Therefore, the main tournament matches were completed within approximately 60 min. Additionally, teams eliminated from the tournament were encouraged to participate in friendly matches with other eliminated teams during the remaining time or to support their favorite team. This format ensured that all students, whether competing or eliminated, remained engaged in gameplay and the broader class activity.

Before and after the class, participants answered the same set of eight questions from the TDMS to measure short-term changes in mood because of their participation in pickleball play [12]. In addition to the fill-in-the-bubbles questions from the TDMS, students were given the opportunity to write a free response in a section at the end of the questionnaire. Of the 106 surveys, 104 were returned, resulting in a response rate of 98&. Of the 104 surveys, 78 free-response answers were recorded and analyzed.

Table 1 (explained in Appendix A) lists the calculated metrics used in this study and their formulas. Depending on the metric, values can range from −20 to 20. Vitality and stability were calculated using eight questions included in the survey. Using the resulting vitality and stability calculations, pleasure and arousal were then computed. Institutional review board approval was obtained from the author’s research institute (Kyushu Sangyo University, Institutional Review Board, 2022-0020), and all procedures adhered to ethical standards for research involving human participants.

## 3. Results

Data were analyzed using IBM SPSS version 27.0. The mean and standard deviation values were calculated before and after the class. Table 2 presents the descriptive statistics (means and standard deviations) for the overall sample as well as by gender, and Table 3 summarizes the results of the two-way mixed ANOVA, including *p*-values and effect sizes. All effect sizes are reported as partial eta squared. Prior to conducting the mixed-design ANOVA, the assumptions of normality and homogeneity of variances were verified. Because the within-subjects factor (time) included only two levels, the sphericity assumption was not applicable.

For vitality, the mixed ANOVA revealed a significant main effect of time (*F*(1,103) = 4.97, *p* = 0.028, *η*^2^ = 0.046), indicating that vitality increased after participation in pickleball. For arousal, the main effect of time showed a trend toward significance but did not reach the conventional level (*F*(1,103) = 3.42, *p* = 0.067, *η*^2^ = 0.032). For pleasure and stability, no significant main effects of time were observed (pleasure: *F*(1,103) = 2.36, *p* = 0.127, *η*^2^ = 0.022; stability: *F*(1,103) = 0.06, *p* = 0.812, *η*^2^ = 0.001). Across all four mood indices, neither main effects of gender nor time × gender interactions were statistically significant.

These results suggest that short-term participation in pickleball led to a modest but significant improvement in vitality, with other mood dimensions showing positive but non-significant trends. Importantly, the analysis indicates that male and female students experienced comparable mood changes, with no statistically significant gender differences.

Additionally, students were provided with a free-response section at the end of the survey, of which 78 were completed in Japanese. Analyzing these qualitative responses adds another dimension to understanding students’ feelings toward the activity and supports the quantitative analysis presented earlier. In total, 64% (50 of 78) of the responses included “fun” or “good”, suggesting that many students found the activity enjoyable. In addition, 24% (19 of 78) of the responses included one of the following kanji: “relationship”, “cooperation”, or “exchange”, reflecting themes of community and collaboration. Altogether, 79% (62) of 78 responses) included at least one of the five positive terms. Next, we discuss the interpretation of our findings, suggest future research directions, and identify the limitations of our study.

## 4. Discussion

The goal of this study is to determine whether pickleball increases mood and positive feelings, as suggested by the research in the previous sections. We used the TDMS to evaluate whether acute momentary affect and mood changes were significant before and after the activity.

Several patterns emerged after examining the results. However, one key observation is the relatively small effect size compared with the values reported by Sakairi et al. [12]. It should be noted that direct comparisons with Sakairi et al.’s findings are limited, as their study employed individual exercise in a non-competitive format, whereas our design involved a competitive tournament in a classroom setting. A review of their data showed a large discrepancy in the pre-activity baseline mean of vitality [12]. Our study showed an elevated pre-pickleball play baseline mean of 4.75 in vitality. This score is notably higher than the reported vitality baseline mean value of 0.06 found in pickleball participants in Sakairi et al., which is a marked difference given a range in values from −10 to 10 [12].

This discrepancy raises the question of the TDMS when the baseline vitality scores are already high. Because the effect size is partially determined by the magnitude of change compared to variability, a higher baseline may limit the potential for further increase, creating a “ceiling effect”. In contrast, Sakairi et al. reported that participants began with low vitality scores, which provided greater opportunities for post-activity improvement, thus resulting in a larger effect size. Another interpretation could involve the pre-tournament context: anticipation and social motivation may have increased the participants’ initial vitality scores. In contrast to Sakairi’s individual exercise conditions, the tournament setting in our study likely generated pre-activity excitement, which elevated baseline vitality. Therefore, these findings suggest that pre-existing mood and active context are fundamental determinants of mood responses, highlighting aspects of the activity experience that effect size calculations alone cannot fully capture.

This raises considerations about the practical significance of smaller effect sizes when baseline mood states vary. Research on leisure activities suggested that these modest changes are significant. Chen et al. [13] found that leisure satisfaction strongly influences long-term well-being, with a predictive effect of β = 0.148 and a strong mediated effect of β_total = 0.706, 95% CI [0.589–0.795]. This indicates that even small momentary mood improvements, such as those observed in our pickleball study sessions, can result in meaningful psychological benefits over time. From this perspective, the short-term increases in vitality and pleasure observed in our pickleball tournament, although modest in magnitude, represent early-stage affective benefits that contribute to longer-term well-being discussed by Chen et al.

Moving to post-activity statistics, students in our study scored higher in every mood category, such as feeling more energized (vitality), calm (stability), happy (pleasure), and alert (arousal), compared to Sakairi et al.’s study [12]. The elevated scores suggest that participation in a longer team-based activity such as a pickleball tournament may provide more comprehensive mental and emotional benefits for acute momentary data than individual whole-body exercises.

While our participants maintained higher absolute scores throughout, starting and finishing with higher numerical values, the two groups appeared to converge on similar qualitative mental states by the end of their pickleball play. The participants in both studies seemed to achieve comparable improvements in mood, highlighting the universal mental health benefits of physical activity. However, the nature of the activity may still affect emotional outcomes. Heo et al. [11] found that serious leisure activities, such as non-competitive pickleball, were associated with lower depression symptoms in older adults. In contrast, our tournament-style format may have introduced performance pressure, which could have influenced arousal and reduced the emotional gains observed in a relaxed leisure environment.

The mixed-design ANOVA indicated no significant main effects of gender or time × gender interactions across the four mood indices, suggesting that male and female students experienced similar mood improvements from the pickleball tournament. Although descriptive data showed slight numerical differences (e.g., increases in vitality and pleasure among men and modest increases in arousal among women), these patterns were not statistically significant and should therefore be interpreted cautiously.

Qualitative reflections also supported overall positive mood improvements. For example, 11 participants mentioned “winning” in their reflections. Both men and women expressed this sentiment, often in relation to the group’s shared experiences rather than as individual competition. This highlights that the social and collaborative aspects of the tournament are important in shaping mood responses regardless of gender.

While most responses expressed positive sentiments, interpreting them based only on linguistic markers, such as the inclusion or exclusion of specific kanji, is imperfect. For example, if the classification relies strictly on the absence of commonly used positive kanji, approximately 20% of the responses could be labeled as neutral or negative. However, closer examination showed that many of these responses were neither critical nor negative. Comments such as “we won”, although lacking explicit emotional language, still imply satisfaction or success rather than negativity. This shows that a subjective qualitative interpretation is necessary to supplement keyword-based classification when analyzing sentiments.

With this in mind, the only negative response we found was a more measured perspective, noting that active participation was necessary to benefit from the experience. This response suggests that although enjoyable, engaging in activities such as pickleball requires active participation. Enjoying an activity depends not only on being present but also on active engagement.

Although the TDMS does not directly measure enjoyment, approximately 60% of participants explicitly mentioned “fun”, which allows us to infer that many participants enjoyed the activity. As mentioned previously, 79% of the participants used at least one of the five terms: “fun”, “good”, “relationship”, “cooperation”, and “exchange”, which suggests not only participation but also a meaningful and socially enriching experience. This finding is supported by Chen et al. [13], who distinguished between leisure involvement and leisure satisfaction. Their study found that casual participation alone does not necessarily increase well-being; rather, emotional fulfillment and enjoyment derived from pickleball experiences are better predictors of improved mental health outcomes. This research provides evidence for the sentiments of a person who gave a response free of negative sentiments: active participation was the difference between enjoying the activity and not enjoying it. Without active participation, there is little to gain from the experience. However, based on the frequent mention of “fun”, it appears that many students actively participated in the tournament and also found it enjoyable, which allows us to conclude that pickleball participation made students feel happy and fulfilled.

Previous studies have shown that leisure activities involving linguistic and social interactions with others positively affect positive emotions, engagement, and life satisfaction [14]. For example, Heo et al. [15] found that older adults who participate in master sports, a leisure activity characterized by high-intensity linguistic and social interactions, report higher levels of life satisfaction and mental health. In our study, we applied a qualitative interpretive approach to analyze the participants’ free-response comments. The comments reveal that the Japanese words: “relationship”, “cooperation”, and “exchange” are frequently used as reflections of in-game communication and social engagement sentiments that occurred during the class. Interpreting the participants’ language patterns showed that their feelings of connection and collaboration generated through pickleball were central to their experiences, suggesting that the social interaction aspect of pickleball plays an instrumental role in enhancing mood. Together, these results suggest that pickleball participation can positively affect student moods in the short term.

These findings indicate that pickleball can produce psychological benefits comparable to those reported for other forms of physical activity. In this sense, the present study does not claim that pickleball is uniquely superior but rather highlights its value as an accessible and socially engaging sport that can deliver similar mental health benefits. Accordingly, pickleball could be considered a practical option for inclusion in university physical education programs, providing students with both the physiological benefits of moderate-to-vigorous exercise and the psychological benefits of a socially interactive activity.

One limitation of this study was the narrow range of participant demographics. All participants were first-year sports science students from a Japanese university and were likely to include more physically active individuals. Because the teams were formed based on pre-existing seminar groups, prior interpersonal relationships may have influenced social dynamics during class and, in turn, affected the participants’ gameplay experiences and mood responses. This potential influence should be considered when interpreting the findings. In addition, because of shared academic and cultural experiences and interests, their baseline values may differ from those of a more general population. This introduces possible sampling bias as the emotional outcomes may not be generalizable to broader, more diverse college student populations. Moreover, the sample was characterized by a gender imbalance (approximately 70% men and 30% women), which limits the statistical power to detect reliable gender differences. Therefore, any gender-related patterns observed in the descriptive data should be interpreted with caution. However, because Japan has a relatively homogeneous culture, this approach may be considered effective. Future research should recruit participants from a broader range of academic disciplines, class years, and cultural backgrounds to improve generalizability and examine whether mood benefits remain consistent across various populations.

This study has several limitations. First, the activity was conducted within a single 100 min class session, making it difficult to determine whether the observed mood improvements were temporary or sustainable over time. Future studies should incorporate repeated sessions and longitudinal approaches to determine whether these effects persist with continued participation. Additionally, the time of day and tournament format were selected for this study; however, these conditions may not be generalizable to other pickleball experiences. Pickleball activities were conducted within a single class period, which may have been relevant to the observed outcomes. Sakairi et al. [12] reported from an unpublished study that the time of day could influence TDMS distribution, which may skew the results. For example, early afternoon testing could produce different mood baselines than morning or evening testing, which could affect the observed changes in scores. Furthermore, the competitive tournament format may have introduced performance pressure or anxiety compared with the casual or individual leisure exercises used in Sakairi’s study. This difference in format may explain the differences in affective outcomes, especially arousal and pleasure. Future research should consider repeating the studies at different times of day or across multiple sessions to determine whether time-based or repeated exposure effects are valid.

Another important limitation of this study is the absence of a control group. Therefore, it is not possible to determine whether the observed mood changes were caused by pickleball itself or influenced by the tournament format, spectators, or the experience of winning or losing. In particular, victory and defeat may have different effects on mood, as evidenced by participants’ qualitative data mentioning feelings such as “I want to win” or “I want to win next time”. Moreover, because the actual amount of playing time varied depending on whether participants advanced in the tournament or engaged in friendly matches after elimination, the “dose” of activity was not standardized. This heterogeneity in participation may have contributed to variability in mood responses and should be addressed in future studies through controlled playtime or standardized activity exposure. These confounding factors constitute the primary limitations of the current study design. Future research could address these limitations by (i) including control conditions such as a spectator group or non-competitive recreational formats, (ii) analyzing mood responses separately for winners and losers to clarify the distinct psychological effects of competitive outcomes, and (iii) standardizing or accounting for differences in actual playing time to reduce variability in the “dose” of activity. Together, these approaches can provide a more nuanced understanding of the psychological effects of pickleball participation.

Moreover, the competitive tournament format may have introduced additional performance pressure compared with non-competitive or recreational settings, and the timing of the class session (midday) may also have influenced baseline mood, as previous research has suggested that time of day can affect TDMS distributions. In addition, because all mood outcomes were assessed using self-report measures, the results may have been influenced by social desirability bias, particularly in a classroom context where participants were surrounded by peers. Taken together, these limitations highlight the need for future studies that incorporate control groups, compare competitive and recreational formats, consider time-of-day effects, and employ complementary objective measures (e.g., physiological indicators) alongside self-report tools. Such approaches would allow a more comprehensive understanding of the psychological mechanisms underlying pickleball participation and its potential applications in educational and health promotion settings.

## 5. Conclusions

This study investigated the effects of pickleball on short-term mood changes among Japanese university students. Participating students showed a significant improvement in vitality, while arousal and pleasure showed positive but non-significant trends, and stability remained unchanged. No significant gender differences were observed, indicating that male and female students experienced similar psychological responses to the activity. In open-ended responses, strictly positive sentiments such as “fun” or “good” were frequently used, which confirms the enjoyment of the activity and the value of social interaction from a subjective perspective. However, the possibility of a “ceiling effect”, in which the effect size was reduced due to the initially high baseline levels of vitality, was also noted. Given the potential influence of existing social connections and the competitive format on the results, future studies comparing non-competitive formats or different group compositions are desirable. In summary, pickleball is an effective activity for short-term mood improvement and holds promise as a tool to support students’ mental health.

## Figures and Tables

**Table 1:** Explanation and Calculation of the Measured Metrics and TDMS Axes.

	Formula
Vitality	Energetic + Lively − Lethargic − Listless
Stability	Calm + Relaxed − Irritated − Nervous
Pleasure	Vitality + Stability
Arousal	Vitality − Stability

**Table 2:** Means and Standard Deviations of Mood Scores Before and After Pickleball (Overall and by Gender).

Variable	Overall Pre *M* (*SD*)	Overall Post *M* (*SD*)	Male Pre *M* (*SD*)	Male Post *M* (*SD*)	Female Pre *M* (*SD*)	Female Post *M* (*SD*)
Vitality	4.75 (4.37)	5.50 (4.08)	4.51 (4.41)	5.32 (4.19)	5.16 (4.32)	5.74 (3.87)
Arousal	−0.80 (4.35)	−0.06 (4.12)	−1.00 (4.45)	−0.48 (4.19)	−0.35 (4.22)	0.90 (3.93)
Stability	5.54 (3.50)	5.55 (3.39)	5.51 (3.71)	5.80 (3.41)	5.52 (3.02)	4.84 (3.27)
Pleasure	10.29 (6.61)	11.05 (6.28)	10.01 (6.82)	11.13 (6.39)	10.68 (6.14)	10.58 (5.99)

**Table 3:** Results of Two-Way Mixed ANOVA for Mood Scores.

Variable	Effect	*F*(df)	*p*-Value	*η* ^2^
Vitality	Time (Pre vs. Post)	*F*(1,103) = 4.97	0.028	0.046
	Gender	*F*(1,103) = 0.39	0.533	0.004
	Time × Gender	*F*(1,103) = 0.24	0.622	0.002
Arousal	Time	*F*(1,103) = 3.42	0.067	0.032
	Gender	*F*(1,103) = 0.28	0.596	0.003
	Time × Gender	*F*(1,103) = 0.04	0.834	0.000
Stability	Time	*F*(1,103) = 0.06	0.812	0.001
	Gender	*F*(1,103) = 0.02	0.888	0.000
	Time × Gender	*F*(1,103) = 0.81	0.37	0.008
Pleasure	Time	*F*(1,103) = 2.36	0.127	0.022
	Gender	*F*(1,103) = 0.23	0.633	0.002
	Time × Gender	*F*(1,103) = 0.92	0.339	0.009

## Data Availability

The data supporting the findings of this study are available upon request from the corresponding author.

## Appendix A

Vitality refers to increased physical and mental activation as well as enhanced motivation to engage in tasks with heightened alertness, which leads to greater participation and productivity. Stability is a state of calmness and balance with reduced physiological activation, which is beneficial for sustained attention and smoother personal interactions. Pleasure is a positive emotional state reflecting feelings of happiness, enjoyment, and satisfaction. Arousal refers to elevated physiological activation that disrupts focus and task performance, resulting in heightened reactivity, difficulty in relaxing, and strained social interaction.
